# Efficacy of favipiravir treatment for patients with severe fever with thrombocytopenia syndrome assessed with a historical control

**DOI:** 10.1128/aac.01062-25

**Published:** 2025-10-17

**Authors:** Masayuki Saijo, Koichi Izumikawa, Koichiro Suemori, Toru Takahashi, Kunihiko Umekita, Hiroki Ohge, Mariko Hatakenaka, Kenichi Ikeda, Daisuke Himeji, Atushi Yamanaka, Norimitsu Kadowaki, Masafumi Kawamura, Yasuyuki Kakihana, Takuo Ito, Kisato Nosaka, Tsutomu Sakurai, Tomoki Yoshikawa, Takeshi Kurosu, Masayuki Shimojima, Masaki Yasukawa

**Affiliations:** 1 Department of Virology 1, National Institute of Infectious Diseases 13511, Tokyo, Japan; 2 Department of Infectious Diseases, Nagasaki University Graduate School of Biomedical Sciences 200674 , Nagasaki, Japan; 3 Department of Hematology, Clinical Immunology and Infectious Diseases, Ehime University Graduate School of Medicine 38050 , Ehime, Japan; 4 Department of Hematology, Yamaguchi Prefectural Grand Medical Center, Yamaguchi, Japan; 5 Division of Respirology, Rheumatology, Infectious Diseases and Neurology, Department of Internal Medicine, Faculty of Medicine, University of Miyazaki 12952, Miyazaki, Japan; 6 Department of Infectious Diseases, Hiroshima University Hospital 68272, Hiroshima, Japan; 7 Department of Emergency and Critical Care Medicine, Kochi Health Sciences Center 47717, Kochi, Japan; 8 Department of Internal Medicine, Kagoshima City Hospital 38271, Kagoshima, Japan; 9 Department of Internal Medicine, Miyazaki Prefectural Miyazaki Hospital 13610 , Miyazaki, Japan; 10 Department of Internal Medicine, Division of Hematology, Rheumatology and Respiratory Medicine, Faculty of Medicine, Kagawa University 12850, Kagawa, Japan; 11 Division of Internal Medicine, Kochi Prefectural Hata Kenmin Hospital, Kochi, Japan; 12 Department of Emergency and Intensive Care Medicine, Kagoshima University Graduate School of Medical and Dental Sciences 208512 , Kagoshima, Japan; 13 Department of Hematology, NHO Kure Medical Center and Chugoku Cancer Center, Hiroshima, Japan; 14 Department of Hematology, Rheumatology and Infectious Diseases, Kumamoto University Hospital 157728, Kumamoto, Japan; 15 Development Division, FUJIFILM Toyama Chemical Co., Ltd 13417 , Tokyo, Japan; The Children's Hospital of Philadelphia, Philadelphia, Pennsylvania, USA

**Keywords:** favipiravir, severe fever with thrombocytopenia syndrome, SFTS, Bandavirus dabieense, SFTS virus, tick-borne virus infection

## Abstract

Severe fever with thrombocytopenia syndrome (SFTS) is a disease caused by SFTS virus (SFTSV), an RNA virus, and endemic to Asia. The case fatality rate in Japan is estimated to be approximately 27%. Currently, no efficacious drugs are available. Favipiravir (FPV) has the potential to treat SFTS. However, no quantitative studies comparing FPV with conventional treatments have been conducted in the country. We conducted an open-label, interventional study in which patients with SFTS were administered FPV 1800 mg twice on the first day, followed by 800 mg twice daily for nine days. Additionally, we performed a retrospective observational study on patients with SFTS who received the best supportive care (BSC) without FPV treatment by collecting medical data from individuals at the same institutions. Patients aged 20–85 years were recruited, and blood samples were collected on Days 0 (pre-dose), 1, 3, 6, 9, 14, and 27 in the interventional study. Outcomes and laboratory parameters were compared by 1:1 matching using propensity score (PS). Thirty and 78 patients were enrolled in the FPV and the BSC groups, respectively. Twenty-three pairs were identified by PS matching. The risk ratio of fatality for the FPV group compared to the BSC group reduced to 0.500 after matching. Ferritin levels associated with hemophagocytosis severity were significantly different between the FPV and BSC groups on Days 3 to 9 after matching. No concerning serious adverse events were observed in the FPV group.

This interventional study has been registered with the Japan Registry of Clinical Trials as jRCT2080223816.

## INTRODUCTION

Severe fever with thrombocytopenia syndrome (SFTS) is a tick-borne infectious disease caused by the SFTS virus (SFTSV), which was first reported in China in 2011 (1, 2). The virus has been officially named *Bandavirus dabieense*, belonging to the genus *Bandavirus* in the family *Phenuiviridae*, order *Bunyavirales* (3), but the synonymous name SFTSV is still widely used.

Patients with SFTS develop high fever, gastrointestinal symptoms, headache, and myalgia, followed by neurological symptoms, such as deterioration in consciousness and hemorrhagic symptoms. Most patients with severe and/or fatal SFTS also develop pathological conditions, such as hemophagocytic syndrome, multiorgan failure, and disseminated intravascular coagulation (4). Its case fatality rates (CFR) were reported to be 16.2% and 32.6% in China and Korea, respectively (5, 6). The CFRs in patients with SFTS in Japan have also been reported to be around 27% since 2012 (7, 8).

The lifecycle and mechanisms of the sustained transmission of SFTSV in nature have been established in some species of ticks, such as *Haemaphysalis longicornis*, *Amblyomma testudinarium*, and *Haemaphysalis flava*. Outbreaks have been reported annually as indigenous infectious diseases in China, Korea, and Japan in the Asia-Pacific region, where the habitat of the ticks has been confirmed (6, 9–11). SFTS is endemic not only to China, South Korea, and Japan, but also to Vietnam, Thailand, and Taiwan (12–14). SFTS is classified as a category 4 infectious disease according to the Infectious Disease Control Law, in which medical doctors were required to register patients with SFTS with the Ministry of Health, Labor, and Welfare (MHLW) in Japan. Approximately 100 patients with SFTS are registered with the MHLW annually (15).

Favipiravir (FPV), a broad-spectrum antiviral agent against RNA viruses, especially negative-sense and single-stranded RNA viruses, is converted to ribosyl triphosphate by intracellular enzymes, and the triphosphate form of FPV selectively inhibits viral RNA-dependent RNA polymerase (16). FPV exhibits broad-spectrum anti-RNA viral activity both *in vitro* and *in vivo* (17–19). FPV demonstrated therapeutic effects against SFTS in animal infection models (20–22).

A single-arm, non-randomized study to evaluate the effectiveness of FPV in patients with SFTS demonstrated that 4 of 23 patients died, resulting in a CFR of 17.4% (23). It suggested that the CFR decreased by approximately 10% compared with previously reported Japanese epidemiological data (7, 8). In this study, patients were orally administered 1,800 mg FPV twice daily on the first day, followed by 800 mg twice daily for 6 to 13 days. In a randomized controlled trial conducted in China, the mean time from FPV treatment initiation to SFTSV shedding cessation was 5.6 days, which was significantly shorter than that in patients receiving standard supportive care without FPV. Additionally, the adjusted hazard ratio for fatality was also significant (24).

Although the clinical effectiveness of FPV in patients with SFTS has been gradually clarified, no quantitative study comparing it with conventional treatments has been conducted in Japan. To address this proposition, the present study investigated the effectiveness of FPV in Japanese patients with SFTS by comparing them with historical controls using the propensity score (PS) (25).

## MATERIALS AND METHODS

### Study design

The effectiveness of FPV was studied by conducting an open-label, uncontrolled, prospective interventional study because conducting a randomized and controlled trial is difficult, owing to the small number of patients with SFTS in Japan. Because this interventional study was not a randomized study, no data were available on patients with SFTS who were treated with regimens without FPV in the study period. Therefore, a retrospective, non-interventional study (observational study) was conducted to obtain the medical data of patients who received the best supportive care (BSC) without FPV at the institutions that participated in the interventional study.

The interventional study design was reviewed and approved by the institutional review boards of each institution, and the observational study design was reviewed and approved by the ethical committees of each institution. This interventional study was conducted in accordance with the Good Clinical Practice guidelines, and the observational study was conducted in compliance with the Ethical Guidelines for Medical and Health Research Involving Human Subjects.

### Participants

Patients aged 20–85 years who were diagnosed with SFTS by reverse transcription polymerase chain reaction (RT-PCR) or those suspected of having SFTS based on the symptoms and laboratory findings were enrolled in the interventional study. The timing of patient enrollment was at the point of obtaining consent after admission. When a negative RT-PCR of SFTSV was obtained, FPV treatment was discontinued, and the patient was switched to another appropriate and supportive treatment regimen. Patients already in the improvement phase, those treated with ribavirin or dialysis before Day 27, taking the day on which FPV treatment was initiated as Day 0, those with immunodeficiency disorders or taking immunosuppressive drugs except adrenocorticoids, those with serious infections, and those with advanced or terminal cancer were excluded. These exclusion criteria were applied only to the interventional study. All the patients with SFTS aged ≥20 years who were treated with non-FPV regimens from March 2013 to September 2017 at research institutions participating in the interventional study were recruited for the observational study. Day 0 for patients in the observational study was defined as the day of hospitalization, as BSC was initiated on the same day in all patients.

### Procedures and outcomes

Patients in the interventional study were administered 1,800 mg of FPV twice on the first day (Day 0), followed by the administration of FPV 800 mg twice daily from Days 1 to 9 (23). The principal investigators or sub-investigators assessed the outcome, patient symptoms, and vital signs before the treatment on Day 0 (pre-dose), during the treatment period (Days 0–9), and the follow-up period on Days 14 and 27. Blood samples were collected on Day 0 (pre-dose), with the day FPV treatment was initiated considered Day 0; Days 1, 3, 6, 9, 14, and 27 for SFTSV genomic load and inflammatory cytokines; and Days 0 (pre-dose), 3, 6, 9, 14, and 27 for quantification of laboratory parameters. All AEs that occurred until Day 27 were recorded.

In this observational study, patient characteristics, such as age, sex, date of disease onset, outcome, therapeutic drugs used, laboratory findings, and vital signs during admission until Day 27, were collected by the investigators from medical records.

The CFR was compared between the two studies until Day 27 as the primary endpoint. In addition, the laboratory parameters available in both studies were compared. The laboratory parameters assessed included ferritin level, platelet count, and aspartate aminotransferase (AST), alanine aminotransferase (ALT), lactate dehydrogenase (LDH), creatine kinase (CK), and activated partial thromboplastin time (APTT) levels, which have been reported as risk factors for SFTS (7).

### Virological test for SFTSV genome detection and quantification

SFTSV genome was detected with RT-PCR, and the genome load was measured with the quantitative real-time RT-PCR as reported previously (26).

### Cytokine level measurement

Cytokine levels of interferon (IFN)-beta, IFN-gamma, tumor necrosis factor (TNF)-alpha, interleukin (IL)-1beta, IL-6, and IL-10 in serum samples were measured using the following kits: VeriKin-HS Interferon β Serum ELISA (PBL Assay Science, Piscataway, NJ), Quantikine IFN-γ Human ELISA Kit, Quantikine HS TNF-α Human ELISA Kit, Quantikine IL-1β Human ELISA Kit, Quantikine IL-6 Human ELISA Kit, and Quantikine IL-10 Human ELISA Kit (R&D Systems, Minneapolis, MN), respectively.

### Statistical analysis

Patients who consented to participate in either the observational or the interventional study were included in the analyses. For comparison between groups, paired matching using PS was applied to minimize the impact of confounding factors (24). The PS was calculated using a logistic regression model with explanatory variables, including age, sex, weight, time from disease onset to treatment initiation, presence of complications, platelet count, WBC count, and National Early Warning Score (NEWS) categories. To select these explanatory variables, the correlation coefficients between the variables were calculated using Spearman’s rank correlation coefficient, and only one variable was used when the coefficient exceeded 0.7. The PS validity condition was that the bias reduction rate should exceed 70%. For matching, a one-to-one pair greedy matching algorithm without a replacement method was adopted. The caliper was one-fourth of the standard deviation of the logit value of the PS.

The Kaplan–Meier method was used to analyze the survival time until Day 27. A logistic regression model was used to estimate the odds ratios. A mixed-effects model for repeated measures was used to analyze longitudinal data, such as laboratory parameters and inflammatory cytokines. The model included treatment, baseline value, visit day, age, time from disease onset to treatment initiation, and the interaction between drug effects and visit day as prognostic factors. All statistical analyses were performed using SAS software version 9.4 (SAS Institute Inc., Cary, NC). Statistical significance was defined as a two-sided *P*-value of <0.05.

## RESULTS

### Interventional study

**Fig 1:**
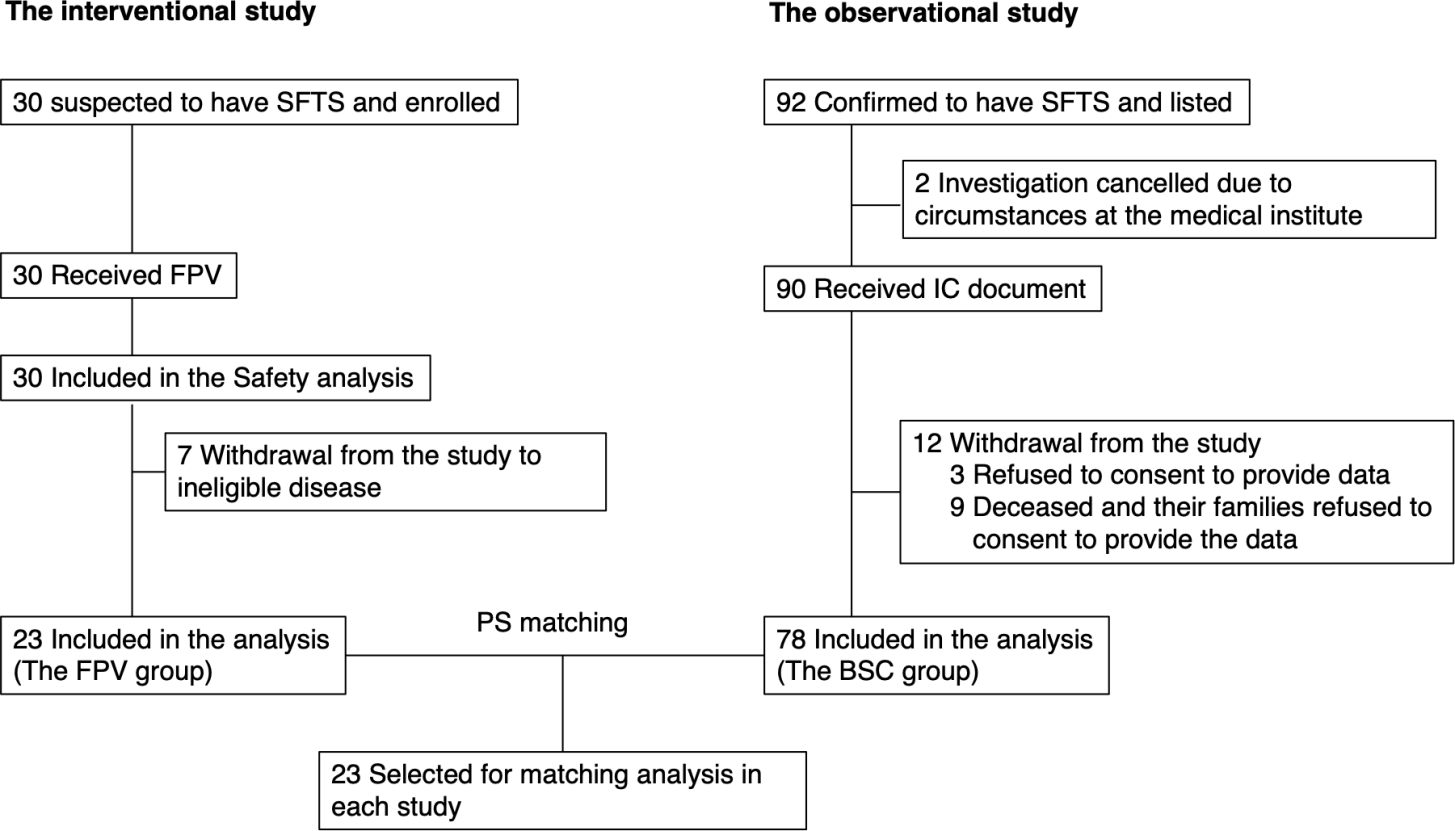
Flow diagram of propensity score matching in both studies. In an interventional study, 30 patients suspected of having SFTS received favipiravir (FPV). Twenty-three patients were confirmed to be infected with SFTSV and were included in the efficacy analysis as the FPV group. In this observational study, 92 patients were diagnosed with SFTS during the study period. Of these, 78 consented to provide their medical data and were included in the efficacy analysis as the best supportive care (BSC) group. After one-to-one greedy propensity score (PS) matching, 23 patients in each group were included in the comparative analysis. IC, informed consent.

Thirty patients with suspected SFTS were enrolled in the interventional study, and all were treated with FPV. Twenty-three patients were subsequently confirmed to be SFTSV-positive and were included in the efficacy analyses as the FPV group (Fig. 1). Of the patients who received FPV, 19 completed the 10-day FPV treatment course. Favipiravir treatment was initiated 2 days before, 1 day before, the same day of, and 1 to 2 days after making virological diagnosis in 1, 13, 5, and 4 patients, respectively.

### Observational study

Ninety-two patients with SFTS who required inpatient care during the study period were identified from the medical records of 18 of the 21 institutions that participated in the interventional study. Two patients declined to participate in the study. Informed consent for data use was obtained retrospectively after follow-up completion from 78 of the 90 patients who were confirmed, based on documented medical records, not to have been treated with FPV. These 78 patients were included in the BSC group. Nine of the 12 patients who declined to provide informed consent for data use were the deceased cases.

Twenty-three participants in the interventional study and 78 participants in the observational study were propensity score (PS)-matched (25), and 23 pairs were identified (Fig. 1).

### Patient background

Approximately three-quarters of the patients enrolled in both studies were patients aged ≥65 years. Among the patient characteristics and baseline laboratory parameters, sex, NEWS category (27), platelet count, ferritin level, AST level, and LDH level differed between the two groups. The proportion of male patients and the baseline levels of ferritin, AST, and LDH in the FPV group were higher than those in the BSC group, whereas platelet counts in the FPV group were lower than those in the BSC group. Although the means of NEWS for both groups were almost the same before PS matching, the proportion of patients who were classified as medium- or high-risk categories in NEWS in the FPV group was higher than that in the BSC group. Patients in the BSC group were distributed almost evenly across each NEWS category. There were no apparent differences in the proportion of sex, mean platelet counts, or WBC counts between the groups by PS matching; however, the distribution of NEWS categories, which were also included in the PS calculation, was still divergent despite matching. The difference in the proportion of patients aged ≥75 years tended to widen after PS matching (Table 1).

**TABLE 1:** Patient demographics and baseline characteristics of SFTS patients in both treatment groups

	Before PS matching		After PS matching	
	FPV*^d^* (*n* = 23)	BSC*^e^* (*n* = 78)	FPV (*n* = 23)	BSC (*n* = 23)
Sex, male, *n* (%)	18 (78.3)	37 (47.4)	18 (78.3)	17 (73.9)
Age, mean (SD*^a^*) years	69.7 (13.5)	71.1 (11.4)	69.7 (13.5)	67.6 (11.7)
<65, *n* (%)	6 (26.1)	22 (28.2)	6 (26.1)	8 (34.8)
65 to 74, *n* (%)	6 (26.1)	23 (29.5)	6 (26.1)	8 (34.8)
≥75, *n* (%)	11 (47.8)	33 (42.3)	11 (47.8)	7 (30.4)
Days from onset*^b^*, mean (SD)	3.8 (2.2)	3.7 (2.9)	3.8 (2.2)	3.7 (1.6)
Platelet, mean (SD) × 10^4^/µL	5.5 (2.9)	6.4 (3.2)	5.5 (2.9)	5.3 (2.0)
Leukocytes, mean (SD)/μL	1,750.0 (1,343.3)	2,005.3 (1,652.6)	1,750.0 (1,343.3)	1,721.3 (1,692.5)
Ferritin, mean (SD) ng/mL	11,138.8 (18,681.5)	6,963.6 (9,012.1)	11,138.8 (18,681.5)	6,578.0 (4,452.8)
AST, mean (SD) U/L	289.5 (319.4)	222.8 (236.5)	289.5 (319.4)	242.7 (240.2)
ALT, mean (SD) U/L	113.4 (98.5)	94.5 (92.2)	113.4 (98.5)	97.6 (64.4)
LDH, mean (SD) U/L	780.9 (511.8)	679.2 (462.0)	780.9 (511.8)	679.1 (418.1)
NEWS*^c^*, mean (SD)	5.7 (2.7)	5.7 (3.5)	5.7 (2.7)	5.8 (3.6)
High (7 to 20), *n* (%)	7 (30.4)	18 (23.1)	7 (30.4)	7 (30.4)
Medium (5 to 6), *n* (%)	11 (47.8)	14 (17.9)	11 (47.8)	4 (17.4)
Low (0 to 4), *n* (%)	4 (17.4)	18 (23.1)	4 (17.4)	7 (30.4)
Unknown	1 (4.3)	28 (35.9)	1 (4.3)	5 (21.7)

^*a*^ SD, standard deviation.

^*b*^ Days from onset for the FPV and BSC treatment groups indicate the interval (days) to the FPV treatment initiation and that to initiation of BSC treatment from the disease onset, respectively. The BSC treatment was initiated from the day of hospitalization in all patients.

^*c*^ NEWS, national early warning score; NEWS is used to predict the outcome of emergency patients, and the higher the score, the greater the risk of poor prognosis.

^*d*^ FPV, favipiravir.

^*e*^ BSC, best supportive care.

### Case fatality rates

Three of the 23 patients (13.0%) were fatal in the FPV group, whereas 14 (17.9%) of the 78 patients were fatal in the BSC group. The risk ratio (RR) for fatalities without PS matching was 0.727. In contrast, the CFR in the BSC group after PS matching increased to 6 of 23 (26.1%). By one-to-one PS matching, the RR and OR of the FPV group were reduced to 0.500 (95% CI 0.142 to 1.762) compared with those of the BSC group (Table 2).

**TABLE 2:** Comparison of case-fatality rate by logistic regression model using PS matching

	Before PS matching		After PS matching	
	FPV*^b^* (*n* = 23)	BSC*^c^* (*n* = 78)	FPV (*n* = 23)	BSC (*n* = 23)
Outcome (*n*, survival/fatal)	20/3	64/14	20/3	17/6
Fatality rate (95% CI*^a^*)	13.0% (2.8–33.6）	17.9% (10.2–28.3)	13.0% (2.8–33.6）	26.1% (10.2–48.4）
Relative risk ratio (95% CI)	0.727 (0.229–2.311)	–^*d*^	0.500 (0.142–1.762)	–

^*a*^ Confidence interval, calculated by Clopper-Pearson method.

^*b*^ FPV, favipiravir.

^*c*^ BSC, best supportive care.

^*d*^ –, although the CFR relative risk ratio decreased, the difference is not statistically significant because of the small number of patients enrolled.

**Fig 2:**
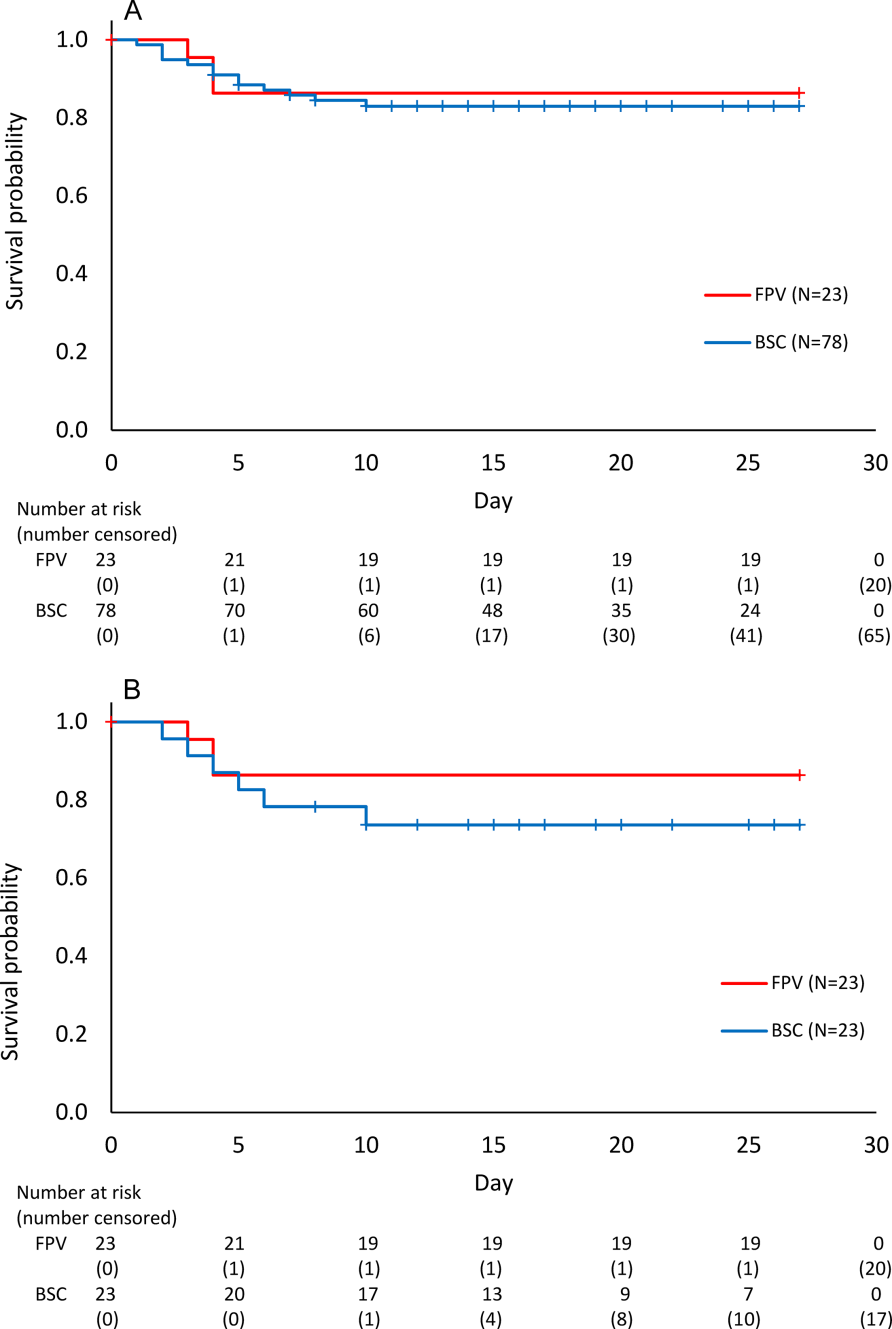
Kaplan–Meier plots of patients with SFTS in both groups before and after PS matching. Survival curves of patients with SFTS in both groups using the Kaplan–Meier method are shown in upper panel (**A**) (before PS matching) and lower panel (**B**) (after PS matching). The red and blue curves represent the FPV and BSC groups, respectively.

From the initiation of FPV treatment, fatal outcomes occurred on Day 2 in one patient and on Day 3 in two patients. None of the patients in the FPV group experienced fatal outcomes on or after Day 4. In contrast, three patients in the BSC group died between Day 4 and Day 9 (Fig. 2).

The CFR of both groups, stratified by high or low fatality risk, according to the prognostic models devised so far—such as NEWS, Wang’s model, and Jia’s model—was compared. In all three models, the CFR in patients with a high fatality risk was higher in both groups than in patients with a low fatality risk. This trend was pronounced in both Wang’s model (28) and Jia’s model (29). No deceased outcomes were observed in patients with a low fatal risk in the FPV group. The CFRs of both models for each risk category were similar. The risk difference was approximately 3% in patients with a low fatality risk and approximately 11% in patients with a high fatality risk, with the risk difference between the two groups expanding in the high-risk category (Table 3).

**TABLE 3:** Comparison of case-fatality rate in SFTS patients stratified by their medical condition

Prognostic model	Fatality risk	Treatment	CFR, n (%)	Difference (95% CI*^f^*)	Relative risk (95% CI*^f^*)
NEWS*^a^*	Low	FPV*^d^* (*n* = 4)	0 (0.0)	−19.0	–^*g*^
	(Score <5)	BSC ^*e*^(*n* = 21)	4 (19.0)	(−50.7 to 12.6)	–
	Moderate or higher	FPV (*n* = 18)	3 (16.7)	−7.5	0.690
	(Score ≥5)	BSC (*n* = 29)	7 (24.1)	(−35.2 to 20.2)	(0.204 to 2.334)
Wang’s model*^b^*	Low	FPV (*n* = 7)	0 (0.0）	−3.0	–
	(Score ≤10)	BSC (*n* = 33)	1 (3.0)	(−17.5 to 11.5)	–
	High	FPV (*n* = 16)	3 (18.8)	−10.8	0.635
	(Score >10)	BSC (*n* = 44)	13 (29.5)	(-38.5 to 16.9)	(0.208 to 1.940)
Jia’s model*^c^*	Low	FPV (*n* = 9)	0 (0.0）	−2.9	–
	(Score ≤0.29)	BSC (*n* = 34)	1 (2.9)	(−15.6 to 9.8)	–
	High	FPV (*n* = 14)	3 (21.4)	−11.1	0.659
	(Score >0.29)	BSC (*n* = 40)	13 (32.5)	(−41.8 to 19.7)	(0.220 to 1.977)

^*a*^ NEWS, national early warning score; NEWS is used to predict the outcome of emergency patients, and the higher the score, the greater the risk of poor prognosis.

^*b*^ Wang’s model = 0.002 × AST + 0.121 × Age + 0.013 × serum Cr (μmol/L).

^*c*^ Jia’s model = 1/{1 + e^- (-14.521 + 0.111 × Age + 0.245 × BUN + 0.089 × APTT)}.^

^*d*^ FPV, favipiravir.

^*e*^ BSC, best supportive care.

^*f*^ Confidence interval (based on chi-square with Yates’ continuity correction).

^*g*^ –, although the CFR relative risk ratio decreased, the difference is not statistically significant, because of the small number of patients enrolled.

### Kinetics of laboratory parameters

**Fig 3:**
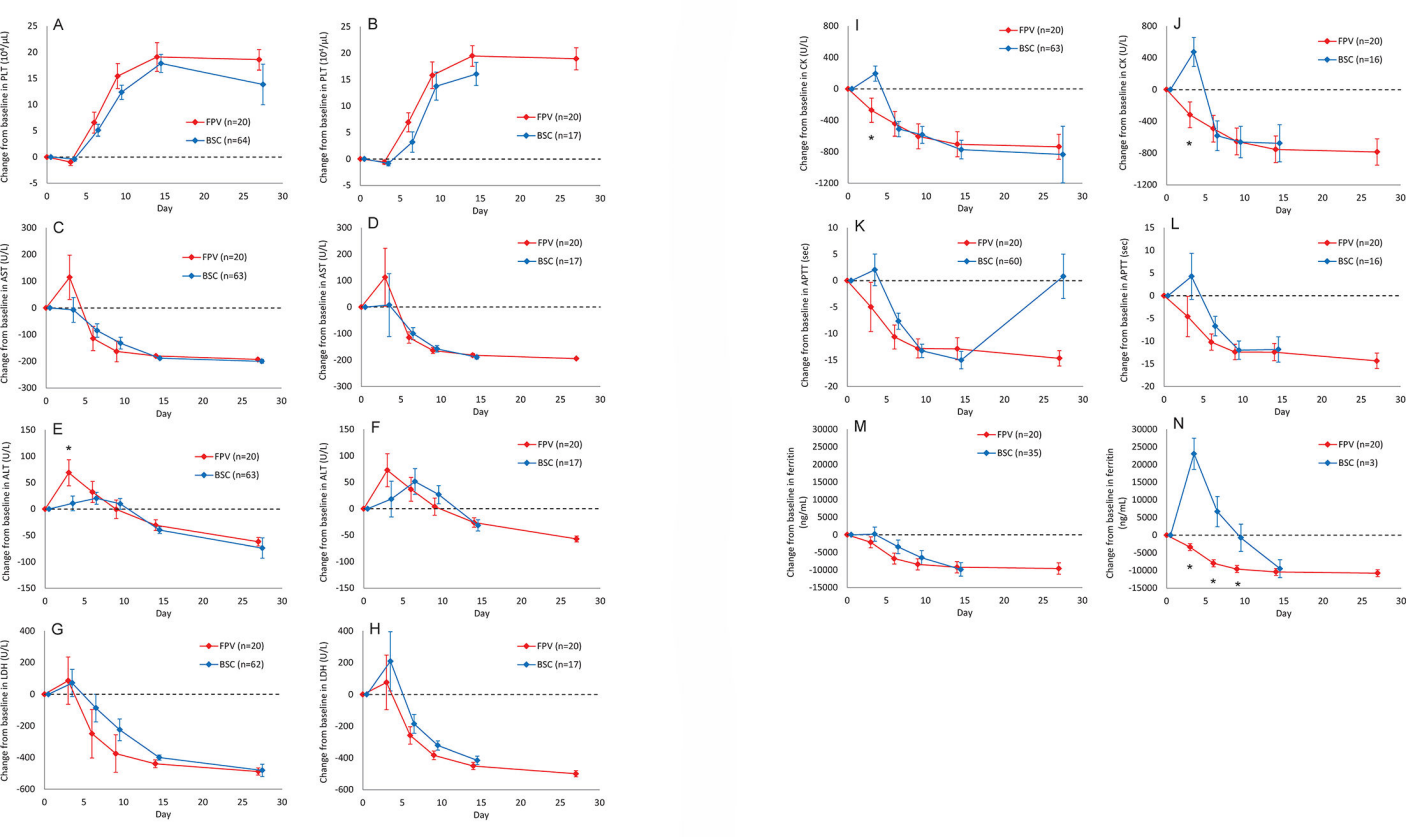
Least square mean (LSM) changes in the laboratory parameters of SFTS after treatment in both groups of survivors before and after PS matching. The adjusted LSM for the changes from baseline in platelet counts and aspartate aminotransferase (AST), alanine aminotransferase (ALT), lactate dehydrogenase (LDH), creatinine kinase (CK), activated partial thromboplastin time (APTT), and ferritin levels for survivors estimated using the mixed-effects models with repeated measures are shown before and after PS matching in panels (**A**) and (**B**), (**C**) and (**D**), (**E**) and (**F**), (**G**) and (**H**), (**I**) and (**J**), (**K**) and (**L**), and (**M**) and (**N**), respectively. The mixed-effects model included treatment, baseline value, visit day, age, time from symptom onset, and the interaction between treatment and visit day as prognostic factors. Red and blue curves represent the FPV and BSC groups, respectively. The vertical lines on each curve represent the standard deviations. Each adjusted LSM with an asterisk indicates a statistically significant difference (*P* < 0.05).

Fatal outcomes were observed in a relatively early phase of disease onset (Fig. 2). In addition, laboratory parameters in fatal cases have the potential to distort the data distribution. Therefore, changes in laboratory parameters of the survivors between the two groups were compared. Platelet counts increased after Day 3 in both groups (Fig. 3A and B). The period until the platelet count increased by more than 100,000/µL was a few days shorter in the FPV group than in the BSC group. An increase in AST from baseline was observed in the FPV group, but not in the BSC group on Day 3, and they decreased after Day 6 in both groups in the observation period without statistical significance (Fig. 3C and D). The kinetics of ALT in both groups were similar to those of AST, although AST levels continued to increase until Day 9 in the BSC group (Fig. 3E and F). The kinetics of LDH did not differ between the two groups; however, the magnitude of the decline was greater in the FPV group than in the BSC group (Fig. 3G and H). The CK levels decreased more rapidly in the FPV group, whereas they increased until Day 3 in the BSC group (Fig. 3I and J), with a significant difference on Day 3 between the FPV and BSC groups (*P* = 0.0108 before matching and *P* = 0.0019 after matching). Similar phenomena were observed in the APTT kinetics (Fig. 3K and L). Ferritin levels, which were abnormally elevated in the very acute phase, also decreased more rapidly in the FPV group than in the BSC group (Fig. 3M and N). In particular, the time course of ferritin levels showed marked differences after PS matching (Fig. 3N). The levels increased until Day 3 in the BSC group, with significant differences between the two groups on Days 3 to 9 (*P* = 0.0000 on Day 3, *P* = 0.0015 on Day 6, and *P* = 0.0293 on Day 9).

### SFTSV viremia kinetics

The mean baseline SFTSV genome load in survivors and fatal cases was 4.1 ± 2.0 (mean ± standard deviation) log_10_ copies/mL and more than 6.0 log_10_ copies/mL, respectively. The least squares mean (LSM) of the change from baseline SFTSV genome load in survivors was 0.2 ± 1.3 log_10_ copies/mL, −1.4 ± 2.0 log_10_ copies/mL, −3.3 ± 2.1 log_10_ copies/mL, and −3.8 ± 1.9 log_10_ copies/mL on Days 1, 3, 6, and 9, respectively. The genome load began to decline on Day 3. Except for one survivor, the genome load declined to approximately the limit of quantification (3.1 log_10_ copies/mL) by Day 6.

**Fig 4:**
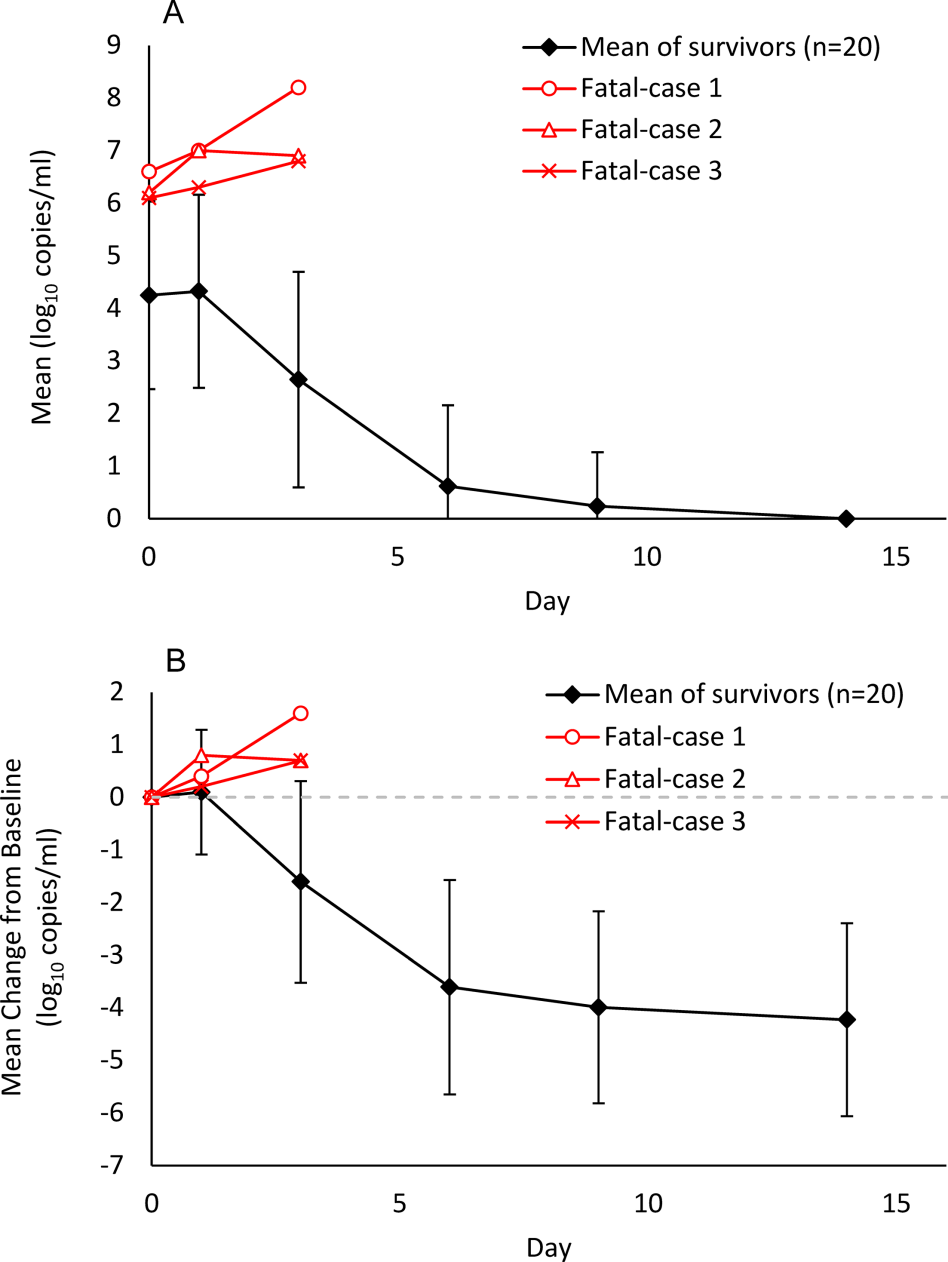
Viral dynamics of SFTSV after FPV treatment in the interventional study. The time course of the SFTSV genome load (**A**) and the change from the baseline viral genome load (**B**) are shown. The viral dynamics of the survivors are presented as the mean of 20 patients. The change from the baseline in the viral genome shown in panel **B** illustrates the least squares mean (LSM) estimated using mixed-effects models with repeated measurements. The mixed-effects model included treatment, baseline value, visit day, age, time from symptom onset, and interaction between treatment and visit day as prognostic factors. The red curve represents each fatal case. The black curve represents the LSM and standard deviation of all the survivors.

In contrast, the LSM of the change from baseline in deceased patients was 0.5 ± 0.3 log_10_ copies/mL and 1.0 ± 0.5 log_10_ copies/mL on Days 1 and 3, respectively. A trend toward an increased SFTSV genome load was observed in deceased patients (Fig. 4).

### Cytokine level kinetics

**Fig 5:**
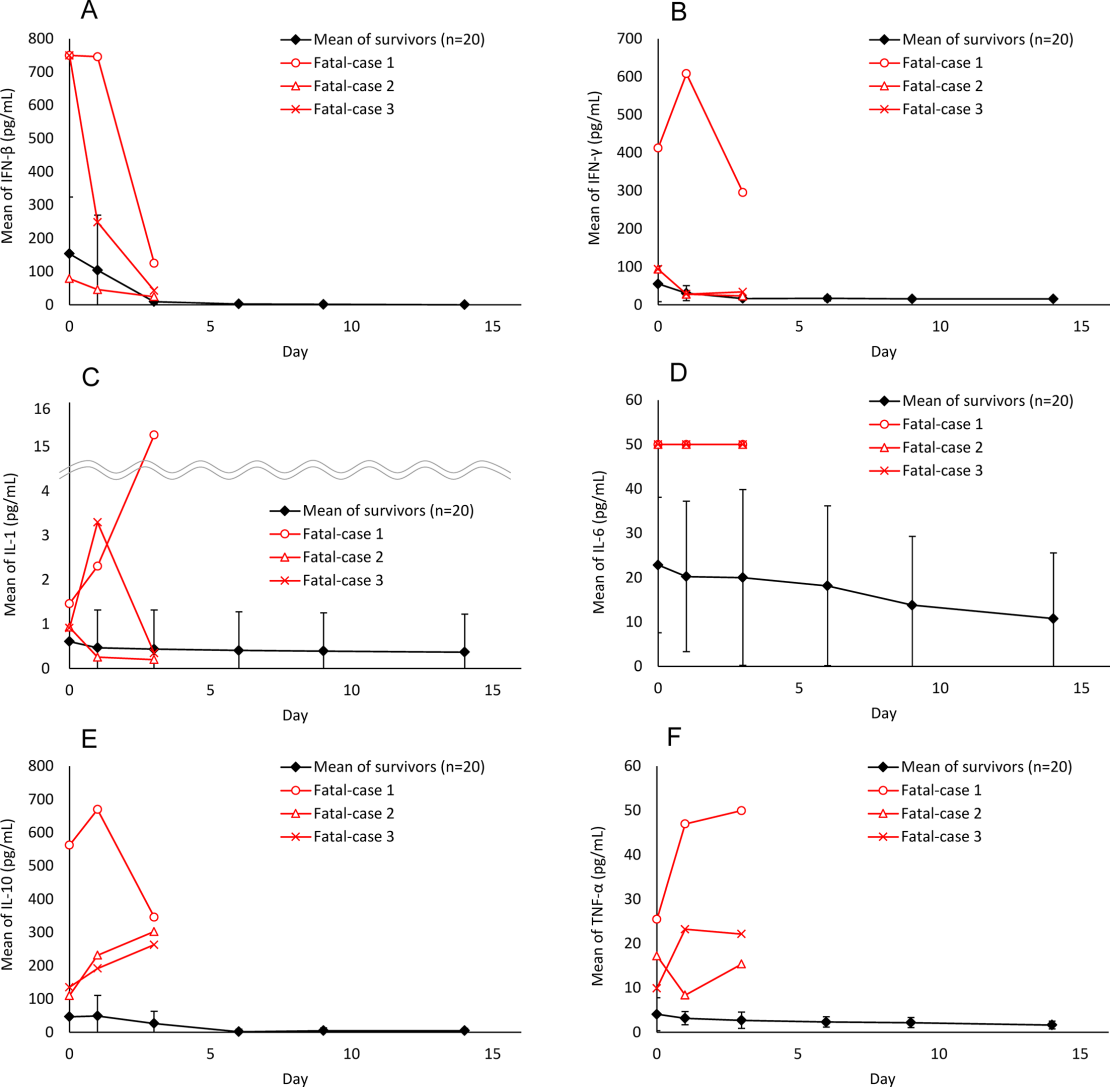
Time course of inflammatory cytokines of patients with SFTS after FPV treatment in the interventional study. The time course of interferon (IFN)-beta, IFN-gamma, interleukin (IL)-1, IL-6, IL-10, and tumor necrosis factor (TNF)-alpha are shown in panels (**A**), (**B**), (**C**), (**D**), (**E**), and (**F**), respectively. The time course of these cytokines in survivors is illustrated as the mean of 20 patients. The red curve represents each fatal case. The black curve represents the LSM and standard deviation of all survivors.

The baseline levels of the inflammatory cytokines examined were generally higher in deceased patients than in survivors. There were clear differences in cytokine kinetics after FPV treatment between deceased patients and survivors. The levels of all cytokines in the survivors decreased, particularly those of IFN-beta (Fig. 5A) and IFN-gamma (Fig. 5B). This trend was also observed in deceased patients, except for IFN-gamma in one patient. The IFN-gamma levels in this patient peaked on Day 1 but decreased by half on Day 3, similar to that in other patients. IL-1 levels in the deceased patients decreased in fatal case 2, peaked on Day 1 and then decreased in fatal case 3, and continued to increase in fatal case 1 (Fig. 5C). Unlike the kinetics of IL-1, IL-6 levels in the deceased patients did not change (Fig. 5D). The IL-10 levels of the two deceased patients continued to increase (fatal cases 2 and 3), whereas those of the other patient peaked on Day 1 and then decreased (Fig. 5E). TNF-alpha levels increased in two deceased patients (fatal cases 1 and 3), decreased on Day 1, and then increased in the remaining deceased patient (Fig. 5F).

### Adverse events

One hundred sixteen adverse events (AEs) were reported in this interventional study. Of these, 40 AEs in 21 of the 30 patients (70.0%) could not be ruled out as being causally associated with FPV treatment. The most frequently observed AEs were hyperglycemia, hyperuricemia, insomnia, and increased blood uric acid levels. Among these frequently observed AEs, the incidence of a causal relationship with suspected FPV was seven (23.3%), six (20.0%), and three of 30 patients (10.0%) for hyperuricemia, increased blood uric acid, and hypertriglyceridemia, respectively (Table 4). Nine serious AEs occurred in eight patients. Of these, serious AEs, which could not be ruled out as causally associated with FPV treatment and corresponded to increased liver function-associated enzymes and neurological symptoms of seizures, were observed in one patient each.

**TABLE 4:** Adverse events (AEs) summary

	Received FPV*^a^* (*n* = 30)		
	No. of patient^*b*^	Incidence (%)	95% CI*^c^*
Overall			
AE	26	86.7	69.3–96.2
AE leading to fatal	3	10.0	2.1–26.5
Serious AE excluded fatal	5	16.7	5.6–34.7
AE leading to FPV discontinuation	4	13.3	3.8–30.7
Adverse events (AEs) for which a causal relationship with FPV cannot be ruled out			
AE	21	70.0	50.6–85.3
AE leading to fatal	0	0.0	0.0–11.6
Serious AE excluded fatal	2	6.7	0.8–22.1
AE leading to FPV discontinuation	3	10.0	2.1–26.5
Frequently observed (at least 3 patients) AEs			
Hyperglycemia	7	23.3	9.9–42.3
Hyperuricemia	7	23.3	9.9–42.3
Insomnia	6	20.0	7.7–38.6
Increased blood uric acid levels	6	20.0	7.7–38.6
Constipation	5	16.7	5.6–34.7
Rash	5	16.7	5.6–34.7
Back pain	4	13.3	3.8–30.7
Oral candidiasis	3	10.0	2.1–26.5
Hypertriglyceridemia	3	10.0	2.1–26.5
Aspiration pneumonia	3	10.0	2.1–26.5
Among the above AEs, for which a causal relationship with FPV cannot be ruled out			
Hyperuricemia	7	23.3	9.9–42.3
Increased blood uric acid levels	6	20.0	7.7–38.6
Hypertriglyceridemia	3	10.0	2.1–26.5

^*a*^ FPV, favipiravir.

^*b*^ Number of patients with at least one case of AE.

^*c*^ Clopper-Pearson confidence interval.

## DISCUSSION

The characteristics of both study populations were similar to those reported so far, which seemed to adequately reflect the characteristics of the patient with SFTS population in Japan (8, 23). The effectiveness of FPV was evaluated by comparing the outcomes of 23 patients treated with FPV in an interventional study with those of an observational study in which the clinical data of 78 patients treated with BSC but not with FPV at 18 medical institutions were collected, retrospectively.

In the interventional study, the CFR up to Day 27 in the FPV group was 13.o% (3 of 23 patients). This is almost similar to the results of 17.4% (4 of 23 patients) reported in a previous study (23). Based on the results from both studies, the CFR for patients with SFTS treated with FPV is estimated to be approximately 15%. The observational study required consent when retrospectively collecting data; therefore, the obtained data tended to be biased toward survivors. Therefore, to enhance comparability, pairs of patients with similar backgrounds were selected from both groups using PS matching. The RR was 0.727 before PS matching, whereas the RR became 0.500 after PS matching. Regarding the time from onset to treatment initiation used as an explanatory variable for PS calculation, the interventional study employed the time from onset to FPV treatment initiation, while the observational study used the time from onset to admission. The mean time from onset to the FPV treatment initiation in the interventional study was 3.8 days. When calculated using the same definition as the observational study, that is, the time from onset to admission, the mean time was 3.5 days in the interventional study. This suggests that the difference in the definition of treatment initiation between the studies had minimal impact.

The effectiveness of FPV in the treatment of patients with SFTS in China using the same PS matching as ours was reported (30). The CFR decreased significantly by introducing FPV treatment, showing an RR for fatality of 0.449. A randomized controlled trial for FPV conducted in China showed that the RR for fatality was 0.517 (24). Based on these results combined with ours, we conclude that FPV treatment reduces the CFR of patients with SFTS by half if initiated in the very early phase of the disease.

The CFR in patients with SFTS in Japan was reported to be higher than that in patients with SFTS in China (5, 8). The reasons and mechanisms for the difference in CFRs have not yet been elucidated. Possible mechanisms include differences in the virulence of SFTSV circulating in nature in each region, test methods, diagnostic capacity, treatment environment, accessibility of patients to medical institutions, and differences in the age distribution of the patients (31, 32). One possible reason is that the proportion of older individuals with SFTS in Japan is higher (11, 15).

To investigate the validity of the results of PS matching, we categorized patients into subgroups according to the magnitude of their fatality risk based on the models and compared the CFR of both treatment groups within each subgroup. The scores of the prognostic models were calculated from the baseline data of patients with SFTS. Wang’s model (28) and Jia’s model (29), in which scores can be calculated from data measured in a relatively large number of patients with SFTS, were selected. The cut-off value was set at 10 in Wang’s model and 0.29 in Jia’s model, with a poor outcome being likely if the patient’s score exceeded these cut-off values. The NEWS is an index mainly developed for triaging emergency patients and classifies scores of ≤4 as low risk, 5 and 6 as moderate risk, and ≥7 as high risk (25). When we classified the condition of the patients in the studies using these prognostic models, the CFR was higher in all the high-risk subgroups. The RR of patients in the FPV group, based on patients with high fatality risk in the BSC group, ranged from 0.635 to 0.690, suggesting the usefulness of FPV for these patients. These results support the robustness of the findings obtained by PS matching.

The clinical laboratory parameters in the survivors of the FPV group tended to improve more rapidly. This tendency was more prominent after PS matching. In particular, ferritin, a diagnostic marker for hemophagocytosis (33), which is often observed in patients with SFTS, showed significant differences from Day 3. The kinetics of ferritin was consistent with a rapid decrease in the levels of inflammatory cytokines in patients in the FPV group. These findings support the notion that FPV treatment alleviated the SFTS-associated symptoms, resulting in an improvement in the CFR.

It is of note that three deceased patients treated with FVP died after inclusion in the investigational study. No bias based on sex assigned at birth was observed in these patients. The baseline values of inflammatory cytokines in these deceased patients were higher than those in the survivors, suggesting that their disease had already progressed with hemophagocytosis and a cytokine storm. All patients were aged ≥80 years and had a high SFTSV genomic load of ≥5 log_10_ copies/mL at baseline. Higher age has been reported to be a risk factor for severe and fatal SFTS (34). SFTSV genomic load has also been reported to differ significantly between fatal and non-fatal cases (26). Furthermore, these patients had secondary bacteremia. Generally, older patients with bacteremia have poor prognoses (35). Therefore, it is necessary to consider the possibility of secondary bacterial infections leading to fatal outcomes in patients who deceased in this study.

Frequently occurring AEs suspected to be causally related to FPV treatment were blood uric acidrelated events (increased blood uric acid and hyperuricemia) and hypertriglyceridemia. AEs related to blood uric acid levels were also commonly observed in patients with COVID-19 administered with the same or lower doses of FPV as in the interventional study (36). This event occurs because FPV and its metabolites inhibit uric acid excretion into the urine via renal transporters and urate transporters (37). This is a reversible reaction. If FPV administration is discontinued, the blood levels of FPV and its metabolites decrease, and the uric acid excretion mechanism returns to normal. Triglyceride levels are significantly higher in patients with SFTS (38). Although the possibility that lipid metabolism disorders are directly associated with SFTS cannot be ruled out, attention should be paid to triglyceride fluctuations when patients are treated with FPV.

Our study had some limitations. First, although the CFR, which was the primary endpoint in the interventional study, was considered to have a relatively low potential for bias contamination because it was a hard endpoint, the investigators were not blinded. Second, the possibility that temporal changes in the SFTS medical environment might have affected the results cannot be ruled out by comparing data from different periods. The data collected in the observational study, which was used as an external control, were not contemporaneous with those of the interventional study. Third, because the exclusion criteria for the interventional study patients were not applied for the observational study patients, we consider that the impact of various biases in both studies may not be eliminated completely. Therefore, we enhanced the reliability for the comparability between the two studies by using the PS matching. Finally, the sample size of the investigations was very small. This is an unsolvable issue, given the low number of patients with SFTS in Japan (15). This often has made subgroup analyses challenging.

In summary, FPV was associated with reduced RR. In addition, FPV treatment might improve the deteriorated inflammatory cytokine responses and laboratory parameters more rapidly when patients with SFTS are treated in the early phases of the disease. Therefore, FPV may serve as a specific therapeutic agent for patients with SFTS.
